# Factors Guiding Clinical Decision‐Making in Genitourinary Oncology

**DOI:** 10.1002/cam4.70304

**Published:** 2024-10-22

**Authors:** Marie Wosny, Stefanie Aeppli, Stefanie Fischer, Tobias Peres, Christian Rothermundt, Janna Hastings

**Affiliations:** ^1^ School of Medicine University of St.Gallen (HSG) St Gallen Switzerland; ^2^ Institute for Implementation Science in Health Care University of Zurich (UZH) Zurich Switzerland; ^3^ Department of Medical Oncology and Hematology Kantonsspital St.Gallen (KSSG) St.Gallen Switzerland; ^4^ Swiss Institute of Bioinformatics (SIB) Lausanne Switzerland

**Keywords:** decision criteria, decision factors, decision‐making, genitourinary oncology, oncology, prostate cancer

## Abstract

**Introduction:**

Clinical decision‐making in oncology is a complex process, with the primary goal of identifying the most effective treatment tailored to individual cancer patients. Many factors influence the treatment decision: disease‐ and patient‐specific criteria, the increasingly complex treatment landscape, market authorization and drug availability, financial aspects, and personal treatment expertise. In the domain of genitourinary cancers, particularly prostate cancer, decision‐making is challenging. Despite the prevalence of this malignancy, there are few in‐depth explorations of these factors within real‐world scenarios. Understanding and refining this intricate decision‐making process is essential for future successful clinical decisions and the integration of computerized decision support into clinicians' workflows.

**Aim:**

The objective of this study is to improve the current knowledge base and evidence of the factors that influence treatment decision‐making for patients with genitourinary cancers.

**Methods:**

Assessment of how routine treatment decisions are made for genitourinary cancers was performed by a mixed‐methods study, encompassing field observations and focus group discussions.

**Results:**

In total, we identified 59 factors that influence clinical decision‐making in oncology, specifically for genitourinary and prostate cancer. Of these, 23 criteria can be classified as decision‐maker‐related criteria encompassing personal, cognitive, and emotional attributes and factors of both, healthcare professionals and patients. Moreover, 20 decision‐specific criteria have been identified that refer to clinical and disease‐related factors, followed by 16 contextual decision factors that describe the relevant criteria introduced by the specific circumstances and environment in which the treatment decision is made.

**Conclusion:**

By presenting an exhaustive set of decision factors and providing specific examples for genitourinary cancers, this observational study establishes a possible framework for a better understanding of decision‐making. Moreover, we specify and expand the set of decision factors, while emphasizing the importance of cognitive, emotional, and human factors, as well as the quality and accessibility of decision‐relevant information.

## Graphical abstract

Clinical decision‐making in genitourinary oncology is a complex process shaped by multiple factors. Decision‐making in routine clinical practice was assessed within a real‐world scenario. Fifty‐nine criteria influence decision‐making, thereof 23 decision‐maker‐related, 20 decision‐specific, and 16 contextual factors. Expanding decision criteria to encompass cognitive, emotional, and human aspects is critical for full comprehension. Ensuring that decision‐relevant information is accessible and of high quality for the entire healthcare team is critical.

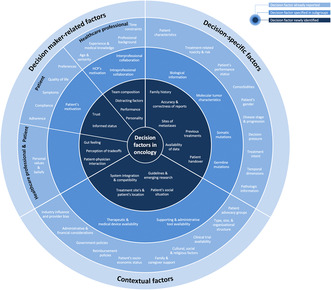

**Abbreviations**
AI: artificial intelligence
CDSS: clinical decision support system
COREQ: Consolidated Criteria for REporting Qualitative Studies
CRPC: castration‐resistant prostate cancer
HCPs: healthcare professionals
HSPC: hormone‐sensitive prostate cancer
mCRPC: metastatic castration‐resistant prostate cancer
mHSPC: metastatic hormone‐sensitive prostate cancer
MSI: microsatellite instability
MTB: multidisciplinary tumor board
PC: prostate cancer
PSA: prostate‐specific antigen
PSMA: prostate‐specific membrane antigen

## 1 Introduction

Clinical decision‐making in oncology is a highly complex process that is influenced by a diverse range of different parameters and stakeholders [1]. The main objective of clinical decision‐making is to identify the optimal treatment type and sequence for a specific cancer patient within a given context [2]. This determination varies based on numerous individual factors and frequently revolves around objectives such as achieving a cure, extending survival, enhancing the quality of life, and alleviating symptoms while preventing adverse treatment effects [3].

Treatment selection is complicated and physicians often face a high amount of decisional conflicts and state uncertainty when making treatment decisions in cancer care [4, 5]. Firstly, the choice of therapy is influenced by a wide range of criteria that are incorporated into every decision‐making process, including clinical factors, patient‐related considerations, and contextual configurations [5, 6]. Secondly, the increasing number and type of cancer treatments introduce complexity into decision‐making [5]. In the field of oncology, traditional treatment modalities like surgery, radiation therapy, and chemotherapy may be administered either individually or in combination. Meanwhile, contemporary modalities extend from hormone therapy to targeted therapy, or cell‐based therapies, with each therapeutic intervention leading to distinct outcomes and challenges, thereby complicating the selection process [7]. Thirdly, the effective selection of treatment mandates a multidisciplinary approach, necessitating collaboration among healthcare professionals (HCPs) with complementary skills such as oncologists, surgeons, radiation oncologists, radiologists, and pathologists [8]. Consequently, achieving optimal treatment outcomes demands substantial investments in time and financial resources, and needs sufficient coordination among all stakeholders [9]. Lastly, patient‐centered oncology and the active participation of cancer patients in decision‐making brings various advantages; however, this approach simultaneously places an increased demand on HCPs' time and efforts to engage patients and their caretakers in the decision‐making process [10, 11].

As exemplified in the field of genitourinary oncology, particularly within prostate cancer (PC) treatment, studies highlight the intricate nature of the decision‐making process [12, 13, 14]. This complexity is not solely confined to HCPs but also encompasses patients, who frequently grapple with the daunting task of selecting the optimal treatment option [15, 16]. PC is a condition estimated nearly 1.4 million newly diagnosed cases and 375,000 deaths worldwide, making it the second most frequently diagnosed cancer and the fifth leading cause of cancer‐related deaths among men in 2020 [17]. More specifically, in the case of metastatic PC, various treatments can demonstrate comparable efficacy, introducing uncertainty for patients during the shared decision‐making process [18]. The understanding of diagnosis, staging, and treatments underlies a constant evolution, resulting in frequent updates to the treatment guidelines, which leads to challenges for HCPs, who have limited time to keep up with and access the extensive and vast amounts of information [19]. An additional challenge is the uncertainty and lack of evidence for certain treatment sequencing such as for metastatic castration‐resistant prostate cancer (mCRPC) [20]. Up‐front use of treatments in terms of intensification in metastatic hormone‐sensitive prostate cancer (mHSPC), may pose a challenge due to the lack of evidence for the choice and possible reduction in the effectiveness of subsequent therapies, for instance, due to cross‐resistance between agents [21, 22, 23].

To address these issues, technological advancements, including computerized clinical decision support systems (CDSS) and artificial intelligence (AI), are increasingly applied and can provide individualized recommendations to enhance clinical efficiency and reduce medical errors [24, 25]. As crucial as technical advancements are, the human‐centered design of these technologies [26], which encompasses an understanding of human behavior, social dynamics, and contextual elements, is important for the successful implementation and user adoption of CDSS^26^. Despite the increasing application and integration of these technologies [25], certain limitations persist, with uncertainties surrounding their impact on HCPs, patient outcomes, and associated costs [24]. Additionally, AI‐based CDSSs encounter challenges related to trust, explainability, scalability, and effective deployment [27, 28].

Effectively integrating decision‐aid technologies and gaining a profound understanding of the decision‐making process and its critical factors are crucial steps to optimize decision‐making in oncology, overcome its complexities, and ultimately improve cancer patient care. Previous research has classified common decision‐making criteria in oncology into three distinct categories, including decision‐maker‐related attributes, pertaining to both physicians and patients, decision‐specific criteria, comprising traditional clinical factors, and contextual factors [1]. While global commonalities exist across most of these decision factors, an emphasis is largely placed on the latter in low‐ and middle‐income countries, including a patient's socioeconomic status, reimbursement policies, and access to treatment, often due to heightened resource limitations [29]. Despite the complexity of clinical decision‐making in genitourinary oncology, particularly in the context of PC, and the significant prevalence of this condition, only limited research has been conducted so far [30]. Thus, the objective of this study is to augment the existing evidence by examining the decision factors in genitourinary oncology in a real‐world setting.

### 1.1 Aim of the Study

The aim of this study is to assess the decision‐making processes in a real‐world setting and understand which are influencing treatment selection in routine genitourinary oncology, with a particular emphasis on PC, thereby contributing to the existing evidence in decision‐making and enhancing the current understanding. To achieve this, we conducted a qualitative study that built upon the foundation laid by Glatzer et al. [1], whose narrative review reflects on various aspects of decision criteria in clinical oncology, as well as on the work by Papadakis et al. [31] on strategic decision‐making processes, and the influence of conjectural factors on strategic decision‐making described by Elbanna and Child [32].

## 2 Methods

### 2.1 Study Design and Setting

This cross‐sectional mixed‐methods study encompasses qualitative non‐participant observations with the analysis of semi‐structured field notes and reflexive focus group discussions with clinicians in order to critically examine how decisions are made in the field of genitourinary oncology. This observational method facilitated a comprehensive exploration of naturally occurring behaviors, actions, and events within the real‐world context of clinical decision‐making for cancer treatment selection, contributing distinct benefits to the comprehensiveness and depth of our investigation [33]. Qualitative research employing observational methods has proven effective in providing detailed descriptions of processes within clinical care settings as non‐participant or passive observations enable the researcher to study participants in their natural environments [34].

The study was conducted in a cancer center in Switzerland in the genitourinary oncology department. The reporting adheres to the Consolidated Criteria for Reporting Qualitative Studies (COREQ) checklist (Table S1), a recognized standard for qualitative research [35], as well as the Four‐Dimensional Criteria for evaluating rigor in qualitative research adapted from Lincoln and Guba [36] (Table S2).

### 2.2 Participants and Sampling

The sampled group of HCPs exhibited diversity, comprising physicians and nurses with varying levels of seniority, professional backgrounds, and medical specialties, including medical oncologists, urologists, pathologists, radiation oncologists, and radiologists, who all were actively engaged in the observed routine meetings. Prior to each meeting, the participants were informed about the observation process as part of the consent procedure; however, the specific purpose of the study was deliberately kept vague to minimize the likelihood of physicians altering their behavior systematically in response to the awareness of being observed.

### 2.3 Data Collection

Non‐participant observations were carried out in‐person and on‐site from October 2023 to January 2024, encompassing two multidisciplinary tumor board (MTB) meetings and two routine genitourinary oncology consultation meetings, each involving between 8 and 21 participants, lasting for 1 h each and focusing on discussions pertaining to patients diagnosed with different types of genitourinary cancers. At the time of observation, field notes were taken by one researcher to capture all relevant situational and context information, as well as to document all identified decision factors and all nuances of interdisciplinary collaboration, communication patterns, and the integration of digital tools. Additionally, two 1‐h reflective focus group discussions with medical oncologists from the hospital's local genitourinary oncology department employing open‐ended questions and a presentation of all preliminary results from the observation meetings were conducted with all six authors to comprehensively identify and validate all relevant decision factors. This method facilitated rich dialogue and enabled the exploration of diverse perspectives among the research team, enhancing the depth of understanding regarding the identified decision factors. Operational and theoretical saturation was employed by quantifying the number of decision factors per interview over time, with most codes identified in the initial observation meeting and a decreasing frequency in subsequent meetings. Theoretical saturation was achieved through interactive processes and regular discussions to ensure a comprehensive understanding and interpretation of findings.

### 2.4 Data Analysis

Field notes were analyzed using qualitative content analysis [37] and thematic analysis [38] as an iterative and content‐driven approach by MW and JH, which required re‐reading the notes to identify all relevant decision factors. Deductive thematic analysis was used for the creation of a preliminary codebook of already identified decision factors based on identified literature [1, 31], with prospective coding conducted during or immediately after data collection. The decision factors were analyzed by using ATLAS.ti software [39] and through iterative discussions between MW and JH, codes that represented decision factors were expanded, harmonized, and sorted into three dimensions, and classified in Microsoft Excel, building on the three‐category framework of decision criteria in oncology, including decision‐maker, decision‐specific, and contextual factors [1, 31]. As a next step, all researchers collaboratively reviewed the decision factors to address and resolve any disagreements, ensuring that these factors were meaningfully categorized within the framework.

## 3 Results

### 3.1 Relevant Factors in Clinical Decision‐Making

**FIGURE 1:**
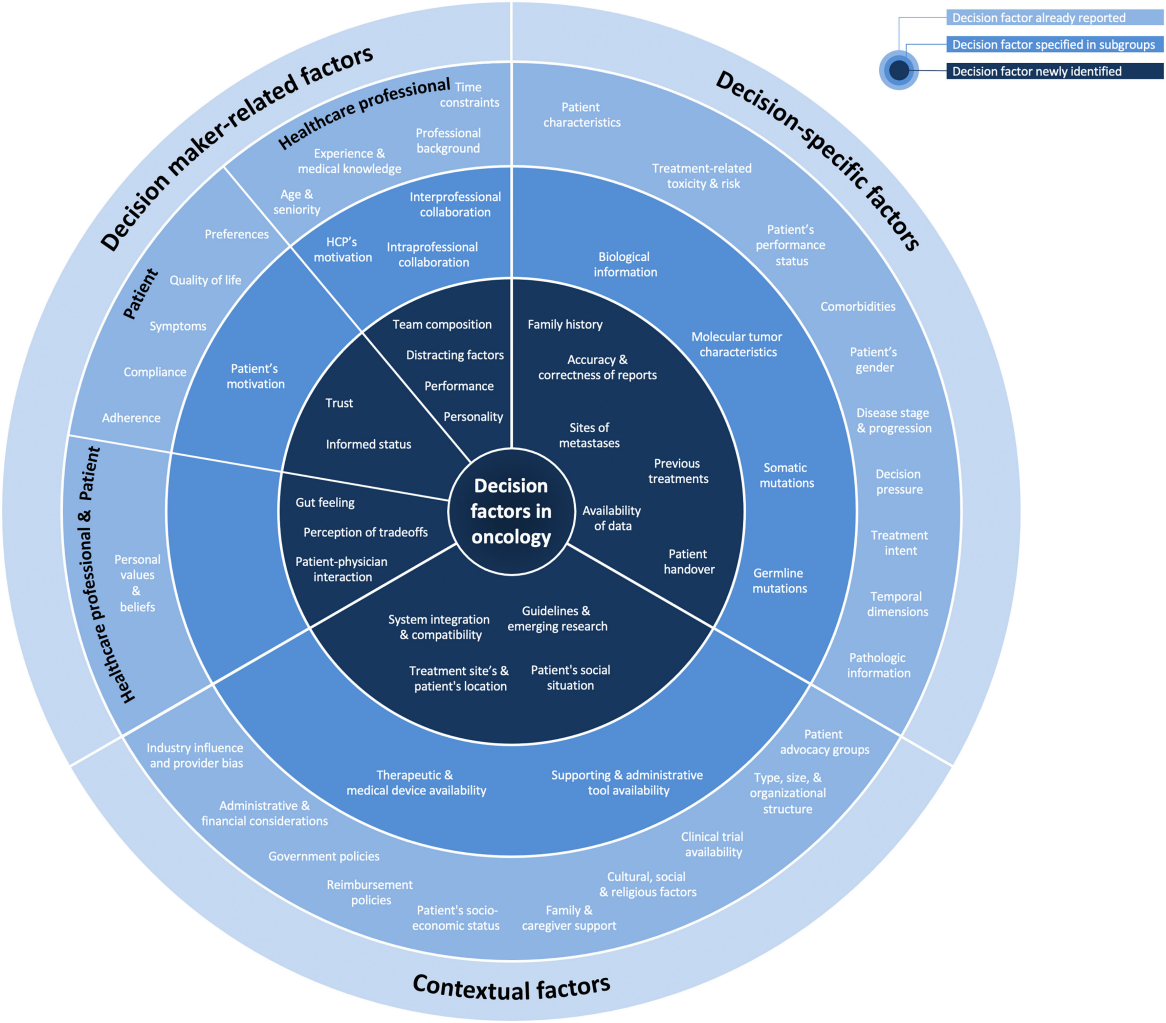
Identified possible decision factors in oncology with a focus on genitourinary oncology, adapted from Glatzer et al. [1].

We identified a total of 59 distinct decision factors for genitourinary cancers, specifically PC, of which we classified 23 as decision‐maker‐related criteria, 20 as decision‐specific criteria, and 16 contextual factors (Figure 1) through focus group discussions and observation meetings (Table S3). Furthermore, we captured specific examples for each of the decision factors.

As compared to the framework established by Glatzer et al. [1], encompassing a total of 34 decision factors, our analysis revealed substantial overlaps with each of these factors across all categories (Table 1). Furthermore, we specified four of the factors into 10 distinct subcategories and uncovered an additional 19 decision factors, distributed among 9 decision‐maker criteria, 6 decision‐specific criteria, and 4 contextual criteria. Therefore, our findings not only propose expansion but also seek to specify certain factors in the field of genitourinary oncology based on real‐world scenarios.

**TABLE 1:** Identified decision factors in genitourinary oncology.

Decision‐maker related factors			Decision‐specific criteria	Contextual factors
Healthcare professional	Patient	Healthcare professional and patient		
Decision factor already reported				
HCPs’ age and seniority level^a^ Work experience and medical knowledge^a^ HCPs’ professional background^a^ Time constraints and workload^a^	Patient's preferences^a^ Quality of life^a^ Disease related symptoms and pain^a^ Patient's compliance^a^ Patient's adherence^a^	Personal values and beliefs^a^	Patient characteristics^a^ Patient's gender^a^ Patient's performance status^a^ Comorbidities^a^ Treatment‐related toxicity and risk^a^ Disease stage and progression^a^ Decision pressure^a^ Treatment intent^a^ Temporal dimensions^a^ Pathologic information^a^	Industry influence and provider bias^a^ Administrative and financial considerations^a^ Government policies^b^ Reimbursement policies^a^ Patient's socioeconomic status^a^ Family and caregiver support^a^ Cultural, social and religious factors^b^ Clinical trial availability^a^ Type, size, and organizational structure^b^ Patient advocacy groups^b^
Decision factor specified in subgroups				
Interprofessional collaboration^a^ Intraprofessional collaboration^a^ HCP's motivation^a^	Patient's motivation^a^		Biological information^a^ Molecular tumor characteristics^a^ Somatic mutations^a^ Germline mutations^b^	Therapeutic and medical device availability^a^ Supporting and administrative tool availability^a^
Decision factor newly identified				
HCPs’ personality^a^ HCPs’ performance^a^ Distracting factors^a^ Team composition and expterise^a^	Patient's trust^a^ Informed status of the patient^a^	Gut feeling^a^ Perception of tradeoffs^b^ Patient‐physician interaction^a^	Family history^b^ Sites of metastases^a^ Previous treatments^a^ Availability of data^a^ Accuracy and correctness of reports^a^ Patient handover^a^	System integration and compatibility^a^ Guidelines and emerging research^a^ Patient's social situation^a^ Treatment site's and patient's location^a^

^a^ Factor observed in meetings and documented in field notes.

^b^ Factor discussed in focus group discussions.

### 3.2 Decision‐Maker‐Related Factors

Decision‐maker‐related factors encompass personal, cognitive, and emotional attributes. These factors pertain to both, HCPs and patients, recognizing the latter as active decision‐makers in their own right with individual needs and interests. This dual perspective emphasizes their individual roles and factors in the decision‐making process and highlights the dynamic interplay between both, which is also relevant for collaborative shared decision‐making.

#### 3.2.1 Healthcare Professional‐Related Decision Factors

##### 3.2.1.1 Decision Factors That Have Been Reported

HCPs' age and level of seniority are critical factors in decision‐making due to their correlation with work experience and clinical judgment, especially having gained experience in different healthcare settings. In addition, medical knowledge, including the knowledge of evidence, guidelines, and interpretation skills regarding diseases, treatments, and side effects, is crucial.

Moreover, HCPs' professional backgrounds have a significant influence, as different medical specialties tend to offer unique perspectives and approaches to patient care. For instance, radiation oncologists tend to concentrate on radiation treatments, while urologists typically focus on surgery [40]. Furthermore, time constraints and heavy workloads are crucial factors, as HCPs often face pressure and limited time for preparation, consultation, and treatment planning.

##### 3.2.1.2 Decision Factors Specified in Subgroups

The factor “professional interaction” can be divided into interprofessional and intraprofessional collaboration. The first describes the collaboration between HCPs from different disciplines, including externals such as general practitioners, with their engagement playing a significant role in ensuring a comprehensive understanding of the patient's history. On the other hand, interprofessional collaboration describes the interaction among HCPs of one healthcare team, which includes their engagement and exchange, but also their capacity to challenge or override decisions and engage in formal collaborations as well as informal discussions. Moreover, the HCP's personal and intrinsic motivation plays a critical role, encompassing dimensions such as altruism toward patient care, financial motivation, or motivation toward shared decision‐making [41, 42].

##### 3.2.1.3 Decision Factors Newly Identified

The composition and expertise of HCPs in a multidisciplinary team often represented in MTBs, is a critical influence on the decision‐making process as the availability and expertise of each individual impact the decision. As the team composition inevitably can change on a week‐by‐week basis, variances in the decision‐making process might be introduced. Other discrepancies may occur due to inconsistent use of medical terminology, such as varied interpretations regarding the tumor status and implications of treatment intensification. In our study, clinicians reported that stage designations such as “hormone‐sensitive prostate cancer (HSPC)” and “castration‐resistant prostate cancer (CRPC)” are not consistently employed across medical specialties, highlighting the need for standardized terminology to ensure clear communication and comprehension among HCPs. Moreover, the personality and performance status of each individual emerge as crucial elements in decision‐making. Their traits and level of openness, engagement, and concentration not only shape the exchange of information but also cultivate a collaborative and insightful environment. Lastly, distracting factors and external stimuli like notifications, emails, and phone calls might influence the decision‐making process, as these interruptions urge HCPs to leave the room and result in a loss of concentration.

#### 3.2.2 Patient‐Related Decision Factors

##### 3.2.2.1 Decision Factors That Have Been Reported

The patient's preference is a primary factor that needs to be respected in treatment selection, encompassing the patient's own choices and desires [43]. Moreover, considering the impact on the overall quality of life for patients before, during, and after treatment is crucial. This consideration extends beyond merely prolonging life, encompassing choices about the type of treatment administration. Moreover, the concept of “time toxicity,” [44] refers to the amount of time spent on therapy and coordinating care, such as visit frequency, travel and waiting times, emergency care for side effects, and hospitalization, which in some cases may outweigh the modest gains in survival. In addition, disease‐related symptoms and pain are crucial considerations to mitigate a patient's discomfort and improve overall well‐being. Additional influences are patient compliance, referring to the passive act of following the recommendations of the HCPs, and patient adherence, reflecting the extent to which a patient willingly and responsibly follows a prescribed treatment based on their capacity and understanding [45], also determining the overall effectiveness of the chosen treatment.

##### 3.2.2.2 Decision Factors Specified in Subgroups

Similar to HCPs, understanding a patient's motivation for a certain treatment involves consideration of internal factors and personal reasons that drive individuals to actively participate in a treatment approach.

##### 3.2.2.3 Decision Factors Newly Identified

The degree of trust a patient has in their HCPs and a chosen treatment significantly shapes therapy selection. This is closely connected to the informed status of a patient and their ability to comprehend the disease and treatment options, as informed patients can better and more actively participate in the decision‐making process, potentially leading to treatment choices that better align with their preferences and values.

#### 3.2.3 The Intersection of Healthcare Professional‐Related and Patient‐Related Decision Factors

##### 3.2.3.1 Decision Factors That Have Been Reported

Decision‐making factors at the intersection of HCPs and patients consider the needs and preferences of both. Personal values and beliefs have a significant impact on decision‐making. This influence is evident in scenarios where HCPs have to weigh whether to prioritize a patient's medical condition over family concerns, a dilemma equally faced by patients.

##### 3.2.3.2 Decision Factors Newly Identified

An individual's gut feeling arises as a subjective yet impactful element, where intuition and personal judgment play a significant role in decision‐making, often complementing rationality and evidence. This is particularly evident in contexts where conventional medical evidence is limited or inconclusive, necessitating clinicians to adapt to uncertainties and rely on their intuitions, drawing from years of experience and tacit knowledge. Equally important is one's perception of trade‐offs between competing objectives, such as maximizing the quantity of life versus minimizing resource utilization, accuracy, or speed, and therefore integral to decision‐making. Finally, the dynamics of patient–physician interaction and communication, whether adopting a paternalistic, HCP‐led approach or embracing a collaborative, shared decision‐making model, significantly shape the decision‐making process and the quality of decisions made.

#### 3.2.4 Decision‐Specific Factors

##### 3.2.4.1 Decision Factors That Have Been Reported

Decision‐specific criteria can be referred to as “classical clinical factors,” which are often highly specialized and require pre‐existing knowledge for complete comprehension. Firstly, patient characteristics, such as age, are integral to decision‐making. Even though younger patients often demonstrate greater tolerance for certain treatment modalities, it needs to be recognized that age alone may not accurately reflect a patient's overall health. Therefore, assessing the patient's performance status is imperative. For instance, evaluating a patient's ability to walk or navigate stairs serves as a vital metric to estimate their capacity to withstand a proposed treatment. This also relates to the consideration of a patient's comorbidities, with the objective of mitigating potential adverse outcomes and minimizing cross‐reactions. Simultaneously, the assessment of treatment‐related toxicity involves a thorough evaluation of potential side effects induced by treatments. This also brings attention to the crucial decision factor of disease stage and progression, which is often reflected by the TNM (tumor, nodes, metastases) classification. For PC in particular, there are further criteria for characterizing the disease, for example, for mHSPC, a distinction is made between high/low volume and high/low‐risk disease, which influences therapy selection and also determines the decision pressure, translated as the urgency of treating a patient, and the pace and intensity of the chosen therapy. In the broader discipline of genitourinary oncology, a patient's gender holds significant relevance, which is not the case for PC.

Another major contributor is the treatment intent, whether directed toward curative measures or palliative approaches, aiming for symptom control and enhancing quality of life. Moreover, temporal dimensions of cancer diagnosis and treatment shape clinical decision‐making. In the case of PC, this involves distinguishing between de novo metastases and metastatic progression after curative local treatment, the time from the diagnosis of HSPC to CRPC. Besides this, pathologic information derived from histology is a determining factor, with particular emphasis on the Gleason score and histological subtype, serving as relevant determinants of the grading and aggressiveness of PC.

##### 3.2.4.2 Decision Factors Specified in Subgroups

A range of clinical factors directly associated with the disease is summarized as biomarkers and laboratory values, which can be further specified. This includes biological information, encompassing blood markers, such as prostate‐specific antigen (PSA) or expression of prostate‐specific membrane antigen (PSMA) that provide valuable insights into the disease aggressiveness and potential treatment responses. Furthermore, molecular tumor characteristics and somatic mutations constitute a diverse set of relevant decision factors, such as microsatellite instability (MSI), which is associated with impaired DNA mismatch repair or mutations impacting signaling pathways, such as *BRCA*. Frequently, increased complexity emerges in instances where patients exhibit unique and rare combinations of mutations, often encompassed with a lack of well‐defined guidelines for making informed decisions. Similarly, germline mutations are relevant as these influence an individual's risk assessment, guide diagnostic testing, and may be the foundation for targeted therapies.

##### 3.2.4.3 Decision Factors Newly Identified

Related to the previous factor, the patient's family history is relevant in the decision‐making not only for the patient but also for relatives, as inherited pathogenic genetic variants, can significantly elevate cancer risk and profoundly affect family members, necessitating collective adjustment through risk management measures and genetic counseling [46]. In the case of PC, treatment decisions are significantly influenced not only by the disease stage and presence of metastases but also by the specific site of metastases. Whether it involves lymph nodes, bones, liver, or lungs, the location of metastases is instrumental for differentiating between prognostically less favorable visceral metastases or bone involvement. Moreover, the significance of a patient's previous treatments and the comprehensive understanding of the spectrum of therapies a patient has undergone is crucial and provides an essential prediction of potential responses, resistances, and overall disease trajectory. This holds particular significance in the context of PC, where resistance to androgen deprivation therapy is a key indicator for disease progression, alongside potential cross‐resistance to later‐stage therapies.

In navigating the intricacies of these decision‐specific clinical factors, ensuring the quality and accessibility of this information is essential for making well‐informed decisions. Hence, the availability of data emerges as another crucial decision factor. Additionally, disparities in data availability and accessibility among members of the HCP team contribute to decision‐making dynamics, which emerge when certain clinicians do not have access to vital information that their colleagues have. Such limitations could stem from technical constraints, such as restricted system access, or time constraints. Likewise, the accuracy and correctness of patient reports are important, as inaccuracies can profoundly affect the quality and reliability of the treatment decision. Also, the process of verbally summarizing the patient's history for patient handovers within healthcare teams or across disciplines plays a substantial role, emphasizing the importance that all relevant patient information is considered and communicated.

### 3.3 Contextual Factors

#### 3.3.1 Decision Factors That Have Been Reported

Contextual factors comprise a diverse range of variables that are crucial elements within a given situation and environment. The field of oncology is intricately shaped by diverse financial and political considerations that can influence treatment selection. One such factor is the influence of industry, marketing, and provider bias including interactions with pharmaceutical sales representatives and manufacturers, who frequently provide complimentary medications. Moreover, administrative and financial factors, such as treatment costs, insurance coverage, licensing, off‐label usage, and the choice between generic and patented treatments are crucial determinants, also extending their influence on diagnostic testing. Another integral aspect in shaping the availability and accessibility of treatments are healthcare systems and government policies. Similarly, reimbursement policies add another layer of complexity to the decision‐making process.

In addition, various contextual factors are based on the patient's individual circumstances. This includes the patient's socioeconomic status, influencing their capacity to afford treatments, manage associated costs, and support services. Equally critical is the role of family and caregiver support, providing essential emotional and practical assistance that can impact the patient's adherence. In line with these considerations, cultural, social, and religious factors are fundamental aspects influencing one's values, beliefs, and preferences, guiding choices not only regarding treatment approaches but also related to end‐of‐life care.

Moreover, clinical trial availability is a core component in decision‐making, offering the opportunity to introduce patients to participate in studies that assess novel treatment approaches. The term “availability” in this context spans from enrollment feasibility, trial site, number of available slots, trial diversity, and individual trial awareness, to knowledge about the respective responsible study nurses. Moreover, the type, size, and organizational structure of a healthcare facility can influence decision‐making, based on varying resources, services, and treatment options. Lastly, patient advocacy groups emerge as a significant influence especially in shared decision‐making, offering information, resources, and support to patients and their caregivers, for instance, the patient‐led, nongovernmental association “Europa Uomo: The European Prostate Cancer Coalition” [47]. These groups can advocate for specific treatments, research, and policies, thereby impacting decisions made by both patients and HCPs.

#### 3.3.2 Decision Factors Specified in Subgroups

Access to resources is a critical factor that can be specified in the accessibility and availability of therapeutics and medical technology as well as the supporting and administrative tools. Access to cutting‐edge therapies and state‐of‐the‐art diagnostic tools can substantially enhance the precision and efficacy of medical interventions, while administrative software allows for more streamlined patient management, optimized resource allocation, and improved communication among HCPs.

#### 3.3.3 Decision Factors Newly Identified

In relation to the previous factors, seamless system integration and compatibility are essential as only well‐integrated and user‐friendly technology tools serve as instrumental support with the potential to elevate the efficiency, accuracy, and comprehensiveness of the decision‐making process. Moreover, treatment guidelines and emerging research, including the frequency and channel through which new results are presented, and subsequently the level to which HCPs keep up with the latest updates, impact the decision‐making process.

Another factor besides the previously mentioned patient's socioeconomic status is the patient's social situation, particularly when they themselves take on caregiving responsibilities for family members, a circumstance often we have observed among older patients. Lastly, the geographical location of the treatment site and its proximity to the patient's residence are fundamental aspects. This encompasses factors such as transportation convenience, potential travel‐related challenges, and associated costs. Consequently, the choice of treatments may be influenced by the practicality of proximity, as stakeholders may navigate decisions considering the impact of distance on accessibility and logistical considerations.

## 4 Discussion

In this study, we aimed to expand and broaden the current evidence of the factors influencing decision‐making in cancer treatment planning, with a specific focus on genitourinary oncology and PC through discussions with and observation of clinicians in a real‐world context.

In total, we identified 59 distinct factors that influence clinical decision‐making in oncology, which, compared to the previous research [1], is an addition of 19 new criteria and 10 specified subgroups. Moreover, we introduced stakeholder perspectives to decision‐maker criteria, addressing the HCPs, patients, or both. The main new factors we observed in our study relate to decision‐maker criteria including team composition and expertise, gut feeling, HCPs' personality and performance status, the informed status of the patient, the perception of trade‐offs, trust, interprofessional collaboration, patient–physician interaction, and distracting factors. We argue that these factors may have been previously underestimated and our observational study provides an ideal setting to explore decision‐making‐related aspects including cognitive, emotional, and human factors that significantly influence the decision‐making process. Other decision factors we observed include decision‐specific criteria, which we propose to specify and expand, based on the field of genitourinary oncology. Even though most of these clinical factors are well known and described in detail [48, 49], we emphasize specifying all decision factors related to biomarkers and laboratory values, and distinguishing between biological and pathological information, molecular tumor characteristics, and somatic as well as germline mutations, considering their distinct testing strategies and requirement of specialized expertise for accurate evaluation.

The key finding of our analysis to emphasize are factors in the decision‐making process that might not be apparent and may have been underestimated in the past. These are related to HCPs and their interaction with each other, their personal motivations, and their courage to disagree and to override previous decisions. Also, external factors lead to distraction and the interruption of the thought process or discussions. Moreover, the daily performance of HCPs, the composition of the team, general time constraints, and workload are also relevant. All of these factors are rather subtle, and it is crucial to raise awareness of them, as these factors might heavily impact a decision.

Lastly, we want to highlight that a large number of decision factors rely on detailed information, and we observed that not only the information itself is critical in the decision‐making process but also the quality of information, and how it is displayed and accessed. In a world where data is becoming more and more abundant, HCPs are constantly required to screen large amounts of information to extract the most relevant [50, 51]. Moreover, a decline in the overall quality of patient reports has been reported in other studies, hence directly influencing the decision‐making process [52, 53]. In addition, seamless access is important for well‐informed decision‐making; however, often this is a main issue in healthcare facilities and also for patients [54, 55]. This challenge extends to situations such as patient handovers within or outside the hospital setting, where critical information may be at risk of being lost [56]. Additionally, we saw that standardizing the understanding of medical terminologies and definitions among HCPs is imperative, as inconsistent use of terms can lead to different understandings. It has been described that inconsistent use and misleading terminology limited clear communication between specialist groups [57], however, a uniform interpretation of words and concepts, is indispensable for promoting a shared understanding as well as effective and cohesive decision‐making [58, 59]. In addition, physician–patient communication is known to be essential in cancer treatment and numerous barriers have been identified related to education and a patient's culture [60]. A study revealed that patients frequently struggle to comprehend medical terminology like “benign,” or “malignant,” highlighting the need for routine educational integration during consultations to empower patients in making informed decisions [61].

Our study reveals the numerous decision factors in treatment selection and therefore highlights that the choice of treatment is not solely determined by medical evidence but is profoundly influenced by individual subjective human factors and contextual elements, which have been described in other studies [62, 63]. Decision‐making in individual settings can differ significantly from decision‐making within a group context, affecting not only the medical field but also various other areas. While individual decision‐making is shaped by personal judgments and experiences, group decision‐making benefits from diverse perspectives and consensus, which can influence treatment choices [64, 65]. Our study focuses on understanding these dynamics in a real‐world setting where group decision‐making processes are at play, highlighting how clinicians collectively contribute to the complex interplay of factors influencing treatment decisions. Therefore, it is evident that a structured approach to decision‐making needs to acknowledge and consider these factors.

This raises the question when it comes to CDSS which of these factors are and can be considered in the design and implementation of these systems. A study showed that CDSS often lack patient‐specific decision factors and patient‐specific input options, and also lack patient‐centered terminology standards, leading to patient and HCP distrust [66]. Other research emphasized that barriers to CDSS adoption are lack of applicability due to a limited number of conditions addressed or patient factors included [67]. Therefore, it is crucial to consider the most prevalent decision factors and studies already highlighted the importance of human factors in CDSS design HCPs should be involved in the development and implementation of CDSS at any stage to provide expert knowledge and ensure clinical workflow integration and consideration of important decision factors [68, 69].

Our study has several strengths but also limitations. Firstly, it provides an in‐depth exploration of decision factors, recognizing their intricate interconnections, which acknowledges the nonlinear and nuanced nature of decision‐making in oncology, contributing to a more comprehensive understanding. However, certain limitations need to be addressed. Firstly, limitations of the qualitative component of this study include that only one researcher took field notes in this study, which ensures consistency and reproducibility, yet limits the breadth of perspectives and may potentially introduce individual biases. Another potential limitation of our qualitative study is the absence of an external audit, which could have provided additional feedback and validation of our findings. While our internal processes ensured rigor, an external review might have further enhanced the accuracy and robustness of our analysis. Secondly, the classification of decision factors may not be entirely mutually exclusive, as their influences on each other can be multifaceted and complex. Moreover, while we successfully reported and observed various patient‐related decision factors, the absence of direct patient participation necessitates caution in generalizing our findings. The transferability of our observations to the wider patient population should be approached with consideration of this limitation. Future research directly incorporating patients would be invaluable for a more holistic understanding, as patients could provide insights into their unique experiences, preferences, and concerns in the decision‐making process. Thirdly, the exclusive focus on the decision‐making process within a single hospital in Switzerland and a specialized therapeutic area may have introduced a potential limitation regarding the broader applicability of our findings, as healthcare settings can vary significantly, and especially contextual factors may differ across diverse environments. In summary, while our focused ethnographic study enriches the understanding of decision‐making processes and highlights specific decision factors in genitourinary oncology, recognizing these strengths and limitations is crucial for the appropriate interpretation and application of the findings.

In conclusion, decision‐making in cancer treatment is highly complex for all stakeholders involved due to various factors that influence the process. In the field of genitourinary oncology and PC, additional challenges arise due to frequent changes in treatment guidelines and a lack of evidence for many clinical scenarios. In this study, we highlight the variety of decision factors that are relevant in treatment selection that add to the understanding of the decision‐making process and might inform strategies to improve the decision‐making process as well as the development of decision aids such as CDSS.

## Author Contributions

**Marie Wosny:** conceptualization (equal), data curation (lead), formal analysis (lead), investigation (lead), methodology (lead), visualization (lead), writing – original draft (lead), writing – review and editing (equal). **Stefanie Aeppli:** investigation (supporting), validation (equal), writing – review and editing (equal). **Stefanie Fischer:** investigation (supporting), validation (equal), writing – review and editing (equal). **Tobias Peres:** investigation (supporting), validation (equal), writing – review and editing (equal). **Christian Rothermundt:** conceptualization (equal), investigation (supporting), project administration (equal), resources (lead), writing – review and editing (equal). **Janna Hastings:** conceptualization (equal), investigation (supporting), project administration (equal), resources (equal), supervision (lead), writing – review and editing (equal).

## Ethics Statement

This study obtained ethical research approval from the academic institution and was deemed out of scope for national ethics approval.

## Conflicts of Interest

S.A. has received compensation from Bristol Myers Squibb (Advisory Board, institutional), Merck (Advisory Board, Speakers' Bureau, institutional), Pfizer (Advisory Board, Speakers' Bureau, institutional), and Pierre‐Fabre (travel support, institutional). S.F. has received compensation from Ipsen (Advisory Board, institutional), Janssen (Speakers' Bureau, institutional), and Bayer (travel support). T.P. has received travel support from Janssen and AbbVie. C.R. has received compensation from Pfizer (Advisory Board, institutional), Bristol‐Myers Squibb (Advisory Board, institutional), and MSD Oncology (Advisory Board, institutional). M.W. and J.H. declare no conflicts of interest.

## Supporting information


Data S1.

## Data Availability

The data that support the findings of this study are available from the corresponding author upon reasonable request.
