# Supplementary material for: Factors Guiding Clinical Decision‐Making in Genitourinary Oncology
Source: Cancer Med. 2024 Oct 22;13(20):e70304. doi: 10.1002/cam4.70304 (PMC11494402; doi:10.1002/cam4.70304)
Supplement: Supplementary file 1 — Data S1. [file CAM4-13-e70304-s001.docx]

Factors Guiding Clinical Decision-Making in Genitourinary Oncology

Supplementary appendix

Marie Wosny^1,2^, Stefanie Aeppli^3^, Stefanie Fischer^3^, Tobias Peres^3^, Christian Rothermundt^3^, Janna Hastings^1,2,4^

^1^School of Medicine, University of St.Gallen (HSG), St Gallen, Switzerland

^2^Institute for Implementation Science in Health Care, University of Zurich (UZH), Zurich, Switzerland

^3^Department of Medical Oncology and Hematology, Kantonsspital St.Gallen (KSSG), St.Gallen, Switzerland

^4^Swiss Institute of Bioinformatics (SIB), Lausanne, Switzerland

**Table of contents**

[Table 1: Consolidated criteria for reporting qualitative studies (COREQ): 32-item checklist 2](#_Toc172844800)

[Table 2: Four-Dimensional Criteria for Evaluating Rigor in Qualitative Research adapted from Lincoln and Guba 5](#_Toc172844801)

[Table 3: Selection of observations and respective field notes from oncology meetings with emerging decision factors and their classification. 7](#_Toc172844802)

# Table 1: Consolidated criteria for reporting qualitative studies (COREQ): 32-item checklist

Developed from *Tong A, Sainsbury P, Craig J. Consolidated criteria for reporting qualitative research (COREQ): a 32-item checklist for interviews and focus groups. International Journal for Quality in Health Care. 2007. Volume 19, Number 6: pp. 349 – 357*

| **No. Item** | **Guide questions/ description** | **Reporting (description/page)** |
| --- | --- | --- |
| **Domain 1: Research team and reﬂexivity** | | |
| **Personal Characteristics** | | |
| 1. Interviewer/facilitator | Which author/s conducted the interview or focus group? | - Marie Wosny  - Janna Hastings |
| 2. Credentials | What were the researcher’s credentials?  *E.g., PhD, MD* | - Marie Wosny: M.Sc., B.Sc.  - Janna Hastings: Prof., PhD, M.Sc. B.Sc.  - Stefanie Fischer, M.D.  - Tobias Peres, M.D.  - Stefanie Aeppli, M.D.  - Christian Rothermundt, M.D. |
| 3. Occupation | What was their occupation at the time of the study? | - Marie Wosny: Research assistant  - Janna Hastings: Professor - Stefanie Fischer: Medical doctor  - Tobias Peres: Medical doctor  - Stefanie Aeppli: Medical doctor  - Christian Rothermundt: Medical doctor |
| 4. Gender | Was the researcher male or female? | - Marie Wosny: Female  - Janna Hastings: Female  - Stefanie Fischer: Female  - Tobias Peres: Male  - Stefanie Aeppli: Female  - Christian Rothermundt: Male |
| 5. Experience and training | What experience or training did the researcher have? | The research team members who conducted the observations and discussion are experienced in qualitative research in the dimension of healthcare, while the medical doctors are experts in the field or genitourinary oncology. |
| **Relationship with participants** | | |
| 6. Relationship established | Was a relationship established prior to study commencement? | MW and JH had prior relationships with the participants. SF, SA, TP and CR know the participants professionally. |
| 7. Participant knowledge of the interviewer | What did the participants know about the researcher? *E.g., personal goals, reasons for doing the research* | Page 3 |
| 8. Interviewer characteristics | What characteristics were reported about the interviewer/ facilitator? *E.g., bias, assumptions, reasons, and interests in the research topic* | Page 3 |
| **Domain 2: Study design** | | |
| **Theoretical framework** | | |
| 9. Methodological orientation and Theory | What methodological orientation was stated to underpin the study? *E.g., grounded theory, discourse analysis, ethnography, phenomenology, content analysis* | Page 3 |
| **Participant selection** | | |
| 10. Sampling | How were participants selected? *E.g., purposive, convenience, consecutive, snowball* | Page 3 |
| 11. Method of approach | How were participants approached? *E.g., face-to-face, telephone, mail, email* | Page 3 |
| 12. Sample size | How many participants were in the study? | Page 3 |
| 13. Non-participation | How many people refused to participate or dropped out? Reasons? | N/A |
| **Setting** | | |
| 14. Setting of data collection | Where was the data collected? *E.g., home, clinic, workplace* | Page 3 |
| 15. Presence of non-participants | Was anyone else present besides the participants and researchers? | Page 3 |
| 16. Description of sample | What are the important characteristics of the sample? *E.g., demographic data, date* | Page 3 |
| **Data collection** | | |
| 17. Interview guide | Were questions, prompts, guides provided by the authors? Was it pilot tested? | N/A |
| 18. Repeat interviews | Were repeat interviews carried out? If yes, how many? | N/A |
| 19. Audio/visual recording | Did the research use audio or visual recording to collect the data? | N/A |
| 20. Field notes | Were ﬁeld notes made during and/or after the interview or focus group? | Page 3 |
| 21. Duration | What was the duration of the interviews or focus group? | Page 3 |
| 22. Data saturation | Was data saturation discussed? | Page 3 |
| 23. Transcripts returned | Were transcripts returned to participants for comment and/or correction? | N/A |
| **Domain 3: analysis and ﬁndings** | | |
| **Data analysis** | | |
| 24. Number of data coders | How many data coders coded the data? | Page 3 |
| 25. Description of the coding tree | Did authors provide a description of the coding tree? | Page 3 |
| 26. Derivation of themes | Were themes identiﬁed in advance or derived from the data? | Page 3 |
| 27. Software | What software, if applicable, was used to manage the data? | Page 3 |
| 28. Participant checking | Did participants provide feedback on the ﬁndings? | Page 3 |
| **Reporting** | | |
| 29. Quotations presented | Were participant quotations presented to illustrate the themes/ﬁndings? Was each quotation identiﬁed? *E.g., participant number* | N/A |
| 30. Data and ﬁndings consistent | Was there consistency between the data presented and the ﬁndings? | Page 3-8 |
| 31. Clarity of major themes | Were major themes clearly presented in the ﬁndings? | Page 3-8 |
| 32. Clarity of minor themes | Is there a description of diverse cases or discussion of minor themes? | Page 3-8 |

# Table 2: Four-Dimensional Criteria for Evaluating Rigor in Qualitative Research adapted from Lincoln and Guba

Adapted from *Lincoln, YS. & Guba, EG. (1985). Naturalistic Inquiry. Newbury Park, CA: Sage Publications.*

| Rigor criteria | Purpose | Technique | Technique applied in this study |
| --- | --- | --- | --- |
| Credibility | Internal validity: Ensures findings accurately represent participants’ perspectives and experiences | Prolonged engagement | We implemented prolonged engagement by spending extensive time within the oncology department of the hospital, immersing ourselves in the clinical decision-making process. This approach enabled us to build rapport with healthcare professionals, understand the specific context of oncology practice, and detect potential distortions in data related to clinician behavior and decision-making dynamics. |
|  |  | Presentient observation | In our study persistent observation was utilized to focus on the most relevant elements of the decision-making process. By concentrating on specific aspects of oncology practice and interactions among healthcare professionals, we gained a detailed understanding of the factors influencing clinical decisions, thereby providing depth to our analysis. |
|  |  | Peer debriefing | We conducted peer debriefing by discussing our analysis inf focus-group discussions to critically examine implicit assumptions and biases related to decision-making factors. This process facilitated the testing and validation of our emergent hypotheses about clinical choices and allowed us to refine our interpretations of how various factors influence decision-making in the genitourinary setting. |
|  |  | Referential adequacy | Data were collected and analyzed concurrently over a four-month period. To validate our preliminary findings, we reexamined portions of the data that had been analyzed previously, ensuring that our conclusions were robust and consistent. |
| Confirmability | Objectivity: Verifies findings are based on data, not researcher bias | Reflexivity | Reflexivity was maintained by recognizing that the analysis was primarily conducted by authors, who are not physicians and have no experience in making clinical decisions. To address potential biases and ensure the accuracy of our interpretations, the findings were validated and discussed with other authors who are oncologists, providing a critical perspective from clinical experts. |
|  |  | Triangulation | We employed triangulation by utilizing multiple data sources, including focus-group discussions, and observations of decision-making processes. |
|  |  | **Audit trail** | We maintained an audit trail by meticulously documenting each step of the research process, from data collection to analysis. This detailed record of our data collection, coding decisions, and analytic procedures allowed us to trace the development of our findings on decision factors and ensured transparency and reproducibility in understanding how clinical decisions were influenced. |
| Dependability | Reliability: Ensures consistent and stable research process and findings | Inquiry audit | - |
| Transferability | External validity: Assesses if findings are applicable to other contexts or groups | Thick description | We provided detailed descriptions of the clinical settings, participant interactions, and decision-making processes. This rich contextual information allows readers to evaluate the relevance of our findings to other oncological or clinical settings. Additionally, we documented operational and theoretical data saturation in detail, facilitating comparisons with other clinical contexts and enhancing the applicability of our conclusions. |

# Table 3: Selection of observations and respective field notes from oncology meetings with emerging decision factors and their classification.

| **Meeting** | **Observation and field notes** | **Decision factor/s** | **Decision factor classification** |
| --- | --- | --- | --- |
| Multidisciplinary tumor board (MTB) | *The patient presentation starts with age, history, diagnosis, performance status, disease status, including the risk/severity and volume/extent of the tumor), and current or prior therapies, followed by initial thoughts and recommendations from the presenting physician.* | Patient characteristics | Decision-specific |
|  |  | Patient’s performance status | Decision-specific |
|  |  | Previous treatments | Decision-specific |
|  | *The oncologist noted that the patient is responsible for caring for his wife with dementia, which may affect his ability to manage complex treatment regimens.* | Patient’s preferences | Decision-maker:  Patient |
|  |  | Patient’s social situation | Contextual |
|  |  | Family and caregiver support | Contextual |
|  | *Oncologist mentioned that the logistics of treatment might be complicated, noting that the burden of regularly attending radiation therapy sessions could be greater compared to the more manageable schedule of medication.* | Treatment site’s and patient’s location | Contextual |
|  | *After the meeting, some more informal discussion was taken place, even though most clinicians directly left the room* | Intraprofessional collaboration | Decision-maker: Healthcare professional |
|  | *Constant phone notifications and pop-up messages were a source of distraction for some physicians.* | Distracting factors | Decision-maker: Healthcare professional |
| Routine genitourinary oncology consultation | *Patient case presentation based on paper notes, edited with markers, with a comprehensive analysis of T, N and M stage.* | Disease stage and progression | Decision-specific |
|  | *Today, the introduction of new guidelines was discussed, including insights into miRNA’s role in disease spread. A printed sheet outlining these guidelines was provided, with access and knowledge restricted to a single copy.* | Guidelines and emerging research | Contextual |
|  |  | Molecular tumor characteristics | Decision-specific |
|  | *While the abbreviations were clear to the physician with a background in internal medicine and cardiology, they were not as readily understood by all oncologists.* | Work experience and medical knowledge | Decision-maker: Healthcare professional |
|  |  | HCPs’ professional background | Decision-maker: Healthcare professional |
|  | *While the initial treatment decisions were made a year ago and were not clearly documented, one physician recalled the decisions from when they were responsible for handing over the patient.* | Patient handover | Decision-specific |
|  |  | Previous treatments | Decision-specific |
|  | *One oncologist asked if the patient could still join a clinical trial targeting this disease type, specifically whether the trial is still open and recruiting participants, and who the responsible study nurse is.* | Clinical trial availability | Contextual |
|  | *The day and meeting was affected by circumstances related to the oncology congress, with only a limited number of staff available in the coming days.* | Time constraints and workload | Decision-maker: Healthcare professional |
